# Excluded-Volume Effects in Living Cells**

**DOI:** 10.1002/anie.201409847

**Published:** 2014-12-29

**Authors:** David Gnutt, Mimi Gao, Oliver Brylski, Matthias Heyden, Simon Ebbinghaus

**Affiliations:** Department of Physical Chemistry II, Ruhr University BochumUniversitätsstrasse 150, 44801 Bochum (Germany); Max-Planck Institut für Kohlenforschung, Kaiser-Wilhelm-Platz 145470 Mülheim an der Ruhr (Germany)

**Keywords:** biophysics, biosensors, excluded-volume effect, FRET, macromolecular crowding

## Abstract

Biomolecules evolve and function in densely crowded and highly heterogeneous cellular environments. Such conditions are often mimicked in the test tube by the addition of artificial macromolecular crowding agents. Still, it is unclear if such cosolutes indeed reflect the physicochemical properties of the cellular environment as the in-cell crowding effect has not yet been quantified. We have developed a macromolecular crowding sensor based on a FRET-labeled polymer to probe the macromolecular crowding effect inside single living cells. Surprisingly, we find that excluded-volume effects, although observed in the presence of artificial crowding agents, do not lead to a compression of the sensor in the cell. The average conformation of the sensor is similar to that in aqueous buffer solution and cell lysate. However, the in-cell crowding effect is distributed heterogeneously and changes significantly upon cell stress. We present a tool to systematically study the in-cell crowding effect as a modulator of biomolecular reactions.

The interior of the cell is a highly crowded environment with macromolecules occupying up to 40 % of the inner volume of a cell.[1] However, biochemical assays and analytical tools are mainly employed in vitro and in dilute aqueous solution, and rarely take effects of the crowded cellular environment into account. The macromolecular crowding effect is often described as an excluded-volume effect.[2] Experimentally, it can be mimicked in vitro using synthetic macromolecular crowding agents like Ficoll 70, polyethylene glycol (PEG), and dextran.[3] In this way, it was shown that the effect of the reduced accessible molecular volume can stabilize proteins,[4] accelerate protein aggregation,[5] increase enzyme catalysis rate,[4] and cause a compaction of disordered proteins.[6]

The excluded-volume paradigm, however, cannot be readily transferred to the cellular level, as contradictory results of protein stabilities in the cell show. For example, phosphoglycerate kinase (PGK) and the B1 domain of protein G were found to be stabilized, whereas VlsE and chymotrypsin inhibitor 2 were destabilized by cell and cell-like environments.[7] A general stabilization effect as predicted by the excluded-volume theory has not been observed.[2,8]

In this work, we design and utilize a crowding sensor to specifically probe excluded-volume effects with spatio-temporal resolution inside living cells and compare the effects to those of well-established in vitro crowding systems. We used polyethylene glycol (PEG, 10 kDa) as a highly soluble, inert, and biocompatible random-coil polymer (Figure S1), which itself is commonly used as a macromolecular crowding agent.[6] In vitro, PEG has previously been shown to be specifically sensitive to the excluded-volume effect.[9] Neutron scattering experiments indicate that it is compressed by 30 % in terms of the radius of gyration in Ficoll 70 (270 mg mL^−1^).[9] Such a molecular compression is the hallmark of the excluded-volume effect: due to the restricted conformational space, a more compact conformation is favored by the macromolecule.[2]

PEG was functionalized by end-group labeling using Atto488 and Atto565 to measure the mean end-to-end distance by FRET (Figure 1 a). We measured a FRET efficiency of (40.3±0.2) % (mean ±s.d.) in vitro (DPBS buffer). The addition and increase in concentration of the macromolecular crowding agent Ficoll 70 caused a compression of the crowding sensor and an increase in FRET (Figure 1 b). As a control, the addition of the monomeric equivalent of Ficoll, the osmolyte sucrose, caused only a minor compression (Figure 1 b). Similar behavior is found for PEG. The macromolecular crowder PEG (10 kDa) leads to a strong compression, whereas lower-molecular-weight oligomers (200 Da) and the monomeric equivalent ethylene glycol (EG) only lead to small compactions. We further tested the osmolyte TMAO which is thought to stabilize proteins via a water-mediated mechanism rather than the excluded-volume effect (Figure S2).[10] TMAO induced only minor changes in the sensor compactness and the FRET efficiency. We further investigated the response towards pH and salt (Figure S3) and found no significant changes in the physiological range. Thus, we show that the sensor is particularly sensitive to the macromolecular nature of the crowding agent.

**Figure 1:**
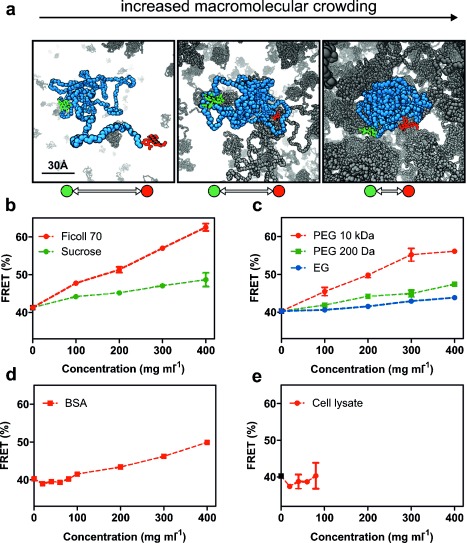
a) Illustration of the labeled PEG sensor in different concentrations of crowding agent (PEG, 10 kDa). Scale bar: 30 Å. b–e) FRET efficiencies plotted as a function of increased cosolute concentration in DPBS buffer for: b) Ficoll 70 and sucrose, c) polyethylene glycol (10 kDa), d) BSA, and e) *Xenopus laevis* oocyte lysate. Error bars represent mean ±s.d.

To mimic the cellular environment in vitro, we tested the response of the sensor towards the protein bovine serum albumin (BSA, Figure 1 d) and a cytoplasmic extract (Figure 1 e) prepared from *X. laevis* oocytes.[11] For both, we found a minor expansion of the sensor at low concentration. At higher concentrations of BSA, the sensor is significantly compressed compared to the expanded conformation at 20 mg mL^−1^. The cell lysate, which could only be concentrated up to a concentration of 80 mg mL^−1^, shows no significant compression effect at its highest concentration.

We then injected the crowding sensor into living HeLa cells (see the Supporting Information for further details). Using pixel-based evaluation we calculated FRET efficiencies on a subcellular scale (Figure 2 a).[12] Surprisingly, we do not find a compression of the sensor, which is expected for physiological concentrations as high as 400 mg mL^−1^ (Figure 2 b). Instead, we find that the FRET efficiency averaged throughout the cytoplasm of a single living HeLa cell is similar to that in dilute aqueous buffer solution. This shows that a counteracting force to the excluded-volume effect must exist in the densely crowded cell. Recent simulations[13] and nuclear magnetic resonance spectroscopy experiments[14] showed that this force could be attributed to weak and transient, nonspecific interactions (e.g. van der Waals and hydrophobic forces) between the crowded macromolecule and the background of crowding molecules. These interactions are proportional to the exposed surface[13d, 15] and thus promote stabilization of expanded chain conformations of PEG, counteracting excluded-volume effects that promote compact conformations. This is in line with the experimental observations that PEG mildly destabilizes proteins through nonspecific binding.[16] The absence of a prevalent excluded-volume effect would explain why on a cellular average, only small and contradictory influences of the cellular environment on protein stability have been found in contrast to the in vitro experiments with polymeric crowding agents.[3,7]

**Figure 2:**
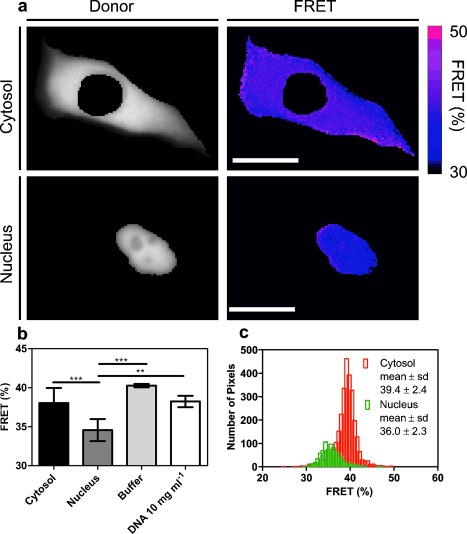
a) Representative donor (Atto488) images of cytosolic and nuclear injected cells. Further, false-colored images of FRET efficiencies calculated for each pixel are shown. Scale bar: 20 μm. b) Comparison of the mean FRET efficiencies in HeLa cytosol (*n*=17), HeLa nuclei (*n*=11), 10 mg mL^−1^ DNA solution (*n*=3), and diluted buffer (*n*=3). One-way ANOVA with multiple comparisons and a Tukey’s post-test were used to determine statistical differences. *P<0.05, **P<0.01, ***P<0.001. The data are reported as mean ±s.d. c) FRET efficiency histogram of the cytosol and nucleus.

However, although the mean differences in the average crowding in the cytosol and in the buffer are rather low, we find that the cellular environment is compartmentalized into differently crowded regions, as can be seen by a broadening of the histogram (Figure 2 c) relative to the in vitro reference which reflects the experimental uncertainty (Figure S4). Even such minor changes in the excluded-volume effect could significantly modulate biomolecular function. It has recently been shown that macromolecular crowding is able to shift the folding equilibrium of a protein by a few kJ mol^−1^ from an unfolded to a folded state.[17] Therefore, distinctively crowded regions could also explain the modulation of the folding landscape of a protein within subcellular environments.[18]

To further explore variations in subcellular crowding inside a cell, we injected the crowding sensor directly into the nucleus. Compared to its conformation in the cytosol and diluted buffer, the sensor is significantly more expanded (Figure 2 b). We show that a simple interaction between DNA and PEG cannot explain this effect by measuring the crowding sensor in concentrated DNA solution in vitro (Figure 2 b). Therefore, the higher expansion of the polymer in the nucleus points towards even lower excluded-volume effects, which could be caused by an overall lower macromolecular content or by the different composition of the nucleus. This finding correlates with faster unfolding rates of PGK in the nucleus which are believed to be caused by a lower local viscosity within the nucleus.[19] Additionally, we find that the nuclear environment, similar to the cytosol, is more heterogeneous than diluted buffer solution indicating a complex crowding environment which is present within different cellular compartments. Besides heterogeneity within cellular compartments, we furthermore observe a variability between the measured cells (Figure S5) for both the cytosolic and the nuclear injected cells. We show that this variability is not caused by concentration differences of the sensor in the cell (Figure S6). Similarly such a cell-to-cell variability was previously observed for protein stability.[19]

One physiologically relevant process in which macromolecular crowding and excluded-volume effects have been proposed to be critically involved is the response of cells to osmotic stress.[20] To test this hypothesis, we exposed microinjected cells to high salt conditions (500 mm NaCl) and therefore increased the macromolecular concentration within the cell, due to the decrease of the cellular volume. We recorded the response to the stress by time-lapsed imaging (Figure 3, Video in the Supporting Information). We show that immediately after the osmotic shock, we observe a strong compression of the crowding sensor. The observed FRET corresponds to a linearly extrapolated Ficoll 70 concentration of approximately 650 mg mL^−1^. Thus, biomolecules in such stressed cells are subject to high compression forces caused by excluded-volume effects. In particular, such forces could act on disordered proteins shown to collapse in crowded environments.[6] Such conditions have also been shown to promote protein aggregation in vitro.[5] Further, crowding could also play a role in the generation of structurally different protein aggregates that have been observed upon osmotic stress.[21]

**Figure 3:**
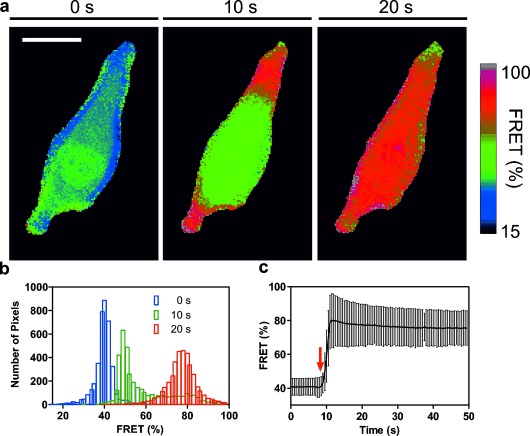
a) Snapshots at three different times during the hypertonic shock are shown: before the hypertonic shock (0 s), immediately after addition of 500 mm NaCl (10 s) and after the hypertonic shock (20 s). Images of the calculated FRET efficiency are shown. Scale bar: 20 μm. b) Histograms of the FRET efficiencies at the three different time points illustrate the subcellular heterogeneity. c) Cell-averaged FRET efficiencies plotted against time. The addition of NaCl is indicated by the red arrow. Error bars represent mean ±s.d.

In conclusion, we have shown that despite the high crowding fraction of the cellular milieu, excluded-volume effects do not significantly compress biomolecules on a cellular average. Our observation can be rationalized by attractive, nonspecific interactions that counteract the excluded-volume effect. However, the osmotic stress experiment shows that under specific conditions excluded-volume effects may play a crucial role in the cellular stress response. The observed cell-to-cell and subcellular heterogeneities suggest that the in-cell crowding effect could further contribute to the spatial regulation of biomolecular function. Future studies will henceforth need to spatially and temporally correlate the physicochemical properties of the cellular environment to a biomolecular probe. Such studies will show whether the cellular environment is an inert “native” matrix in which biomolecules are designed to function or whether it plays an active role in controlling biomolecular processes.
