# Supplementary material for: Excluded-Volume Effects in Living Cells
Source: Angew Chem Int Ed Engl. 2014 Dec 29;54(8):2548–51. doi: 10.1002/anie.201409847 (PMC4506553; doi:10.1002/anie.201409847)
Supplement: Supplementary file 1 [file anie0054-2548-sd1.pdf]

Angewandte Chemie
Eine Zeitschrift der Gesellschaft Deutscher Chemiker

Supporting Information
© Wiley-VCH 2014
69451 Weinheim, Germany

Excluded-Volume Effects in Living Cells**

*David Gnutt, Mimi Gao, Oliver Brylski, Matthias Heyden, and Simon Ebbinghaus**

anie_201409847_sm_miscellaneous_information.pdf

Supplementary Information

S.1 Methods

S.2 Supplementary Figures

S.3 Supplement References

S.1 Methods

S.1.1 PEG FRET sensor

Polyethylene glycol (PEG) with a molecular weight of 10 kDa was synthesized containing amino terminal modifications. Atto488 and Atto565 were conjugated to both ends of the PEG chain. The synthesized PEG sensor was obtained by Rapp Polymere (Tübingen, Germany). Prior to use, PEG sensor was dissolved in DPBS at pH 7.4 (Sigma-Aldrich) in the desired concentrations.

S.1.2 Oocyte extract preparation

Freshly prepared oocytes were kindly provided by the lab of M. Hollmann (Department of Biochemistry I, Ruhr-University Bochum). Isolated oocytes were collagenase treated for 1 h at 20 °C in Ca^{2+}-free Barth's saline to remove the follicle cell layer. The enzymatic reaction was stopped by washing with Ca^{2+}-Barth's saline. Healthy oocytes were selected and used for extract preparation. The extraction was performed similar as described elsewhere.[1] Briefly, oocytes were washed several times with extraction buffer (50 mM sucrose, 100 mM Kcl, 0.1 mM CaCl, 10 mM HEPES-KOH pH 7.7). Cells were transferred to a 2 ml reaction tube and carefully sedimented at 1000 xg. Surplus buffer was removed. 200 µl Nyosil M25 was added on top and then centrifuged at 1000 xg for 1 min allowing for tighter packing of the oocytes. Supernatant oil and buffer was removed and the cells lysed for 15 min at 20,000 xg and 2°C. Translucent yellow cytoplasm was then transferred into new reaction tubes and a 100x protease inhibitor cocktail (Sigma-Aldrich) was added. The mixture was centrifuged at 20,000 xg and 2 °C for 15 min. Again, cytoplasmic fraction was recovered and the protein concentration was determined using Bradford assay with BSA as standard.

S.1.5 Measurement procedure

In vitro and in-cell measurements were performed using the same experimental setup: Measurements were performed at room temperature with fast alternating excitation using a 470 nm and 555 nm LED at 25% power on an AxioObserver Z1 (Zeiss) inverted microscope.

For the measurements, either a 63x/1.2 oil immersion objective (Zeiss) or a 40x/0.95 air objective was used (Zeiss). Excitation light was directed on the probe using a DFT 490+575 (HE) beam splitter (Zeiss). Emission light was guided to a FT 565 (HE) beam splitter (Zeiss) to separate donor and acceptor emission. Donor emission was passed through a BP 512/30 HE filter (Zeiss) and acceptor emission was passed through a BP 630/98 HE filter (Zeiss). Images from donor and acceptor emission were recorded simultaneously using two AxioCam HS (Zeiss) cameras and the AxioVision 4.8.2 (Zeiss) software.

S.1.6 Data analysis

For in-cell data, FRET efficiency was calculated as described by Feige *et al.* using the PixFRET plug-in for ImageJ (NIH).[2] *In vitro* data was evaluated using a self-written Matlab code. For all measurements, background data (either from non-injected cells for cell measurements or from buffer without sensor for *in vitro* measurements) was subtracted separately from each channel. The FRET efficiency was calculated by:[3]

$$FRET = \frac{I_{FRET} - I_{donor} \times Bleed_{donor} - I_{acceptor} \times DE_{acceptor}}{\sqrt{I_{donor} \times I_{acceptor}}}$$

I_{FRET} denotes the intensity of Atto565 with 470 nm excitation, I_{donor} the intensity of the donor with 470 nm excitation, $I_{acceptor}$ the Atto565 intensity with 555 nm excitation, $Bleed_{donor}$ the correction factor for Atto488 emission bleed into the Att565 channel and $DE_{acceptor}$ the correction of direct excitation of Atto565 by 470 nm excitation. Statistical analysis was performed using Graphpad Prism.

S.1.3 *In vitro* measurements

Different crowding solutions (Ficoll70, sucrose, TMAO, PEG 10 kDa, BSA; all Sigma-Aldrich) were prepared at different concentrations in DPBS, pH=7.4 (Sigma-Aldrich) and mixed with the PEG-FRET sensor to yield a final concentrations of 5 µM. For the DNA measurements, ssDNA from *Salmon testis* (Sigma-Aldrich) was used. The solution was placed on glass bottom dishes (fluorodish, WPI) and the measurements were performed with an Axio Observer Z1 (Zeiss) inverse microscope as described before.

S.1.4 Cell culture

HeLa cells were grown in standard T25 culture flasks (Sarstedt) in DMEM media (Sigma-Aldrich) supplemented with 1% penicillin-streptomycin (PS) and 10% fetal bovine serum (FBS) in a humidified atmosphere at 5% CO2 and 37°C. Cells were sub cultured every 2 days

after reaching approximately 80-90 % confluence using standard trypsin digestion and split in a 1:4 to 1:5 ratio into freshly prepared T25 flasks. For experiments, HeLa cells were plated 1 day prior to injection at a density of 2 x 10^5 cells on a 35 mm glass bottom dish.

S.1.5 Microinjection

Microinjection was used to deliver the sensor into HeLa cells using standard protocols.[4] Briefly, HeLa cells were injected 1-2 d after plating. Therefore, the culture media was aseptically removed and exchanged with Leibovitz L15 (Sigma-Aldrich) supplemented with 30 % FBS. Injection was performed using an Eppendorf FemtoJet connected to an Eppendorf InjectMan NI2. FemtoTips II were filled from the back side with a 2 mg ml^{-1} stock solution of sensor and connected to the FemtoJet. The injection parameters were adjusted for each injection so that approximately 5 % of the cell volume was injected. As a starting point, an injection pressure of 100-150 hPa, a compensation pressure of 35 hPa, which causes a constant efflux from the capillary and prevents dilution of the sensor, and an injection time of 0.5 s was used. A few tens of cells were injected within a timeframe of 10 – 15 min. The injected cells were incubated for 10 min at RT to minimize background fluorescence caused from leakage of the sensor from the capillary. Viable cells were imaged within 30 min after injection. Microinjected cells were considered viable when they showed nuclear integrity, adherence to the glass bottom as well as a constant morphology, as reported earlier as criteria for cellular health.[4a,4b,5]

We further showed that cells were viable by nuclear staining using the DNA dye Hoechst 34580 (Sigma-Aldrich). We showed that no changes in adherence, morphology or nuclear integrity were observed before and after injection (Figure S7a). Cells remained viable even 3 h after injection (Figure S7b). Further, nuclear injection did not influence cell viability as shown in Figure S7c. We therefore conclude that the mechanical process of injection does not significantly affect HeLa cell viability in the timeframe of the measurement.

S.1.7 Differential Scanning Calorimetry

DSC was performed using a VP-DSC (MicroCal, Northhampton, USA) with a scan rate of 60 Kh^{-1}. 1 mg ml^{-1} sensor was dissolved in degassed DPBS at pH 7.4 and measured in reference to DPBS buffer. Prior to use, the solution was sterile filtered.

S.2 Supplementary Figures

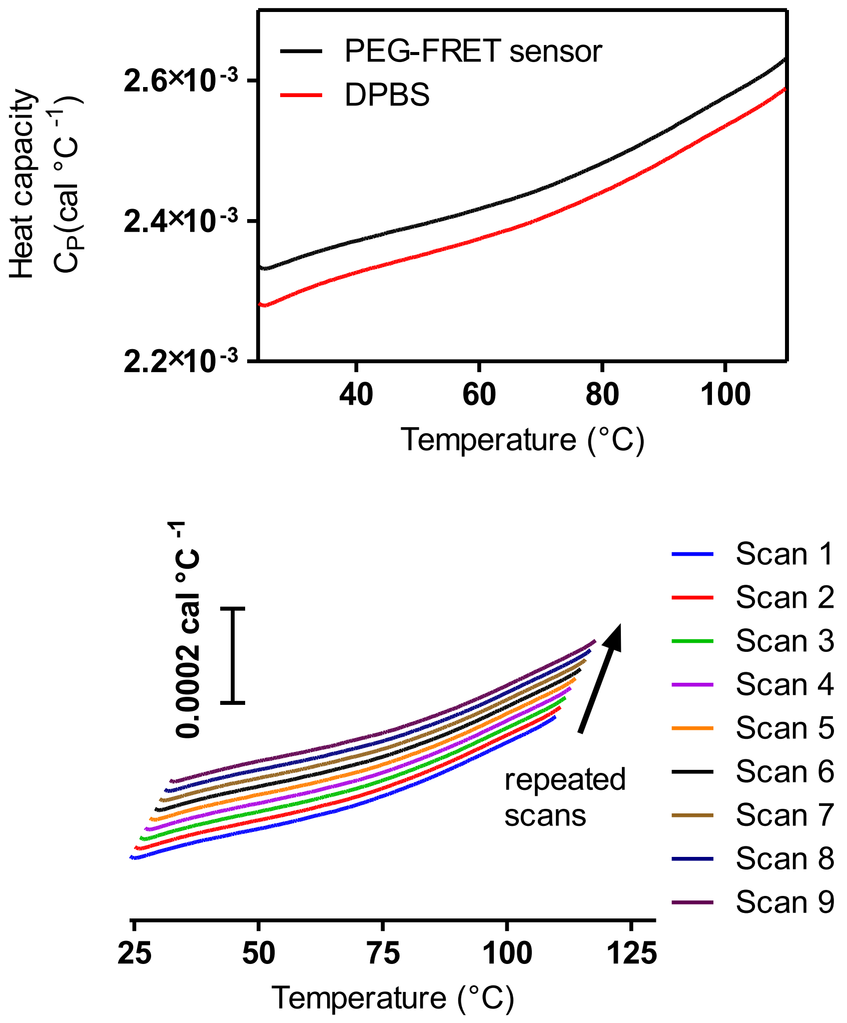

Figure S1 Differential scanning calorimetry of the PEG sensor in DPBS. No specific signatures or transitions in heat capacity were observed. Repeated scans show complete reversibility.

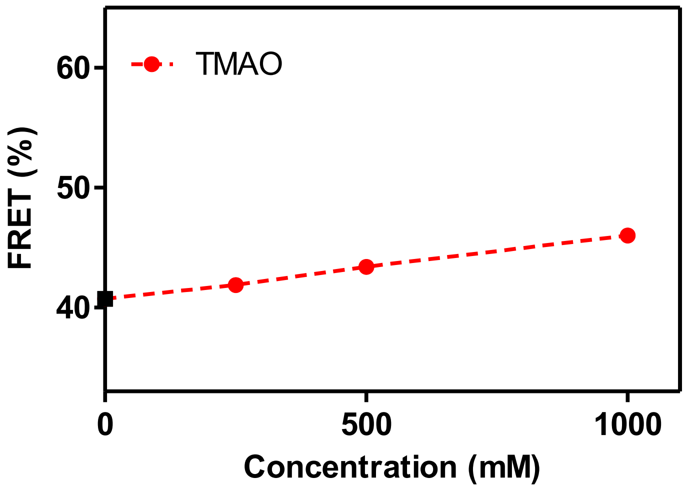

Figure S2. FRET efficiencies measured *in vitro* as a function of increased TMAO concentration. Error bars represent mean ± s.d.

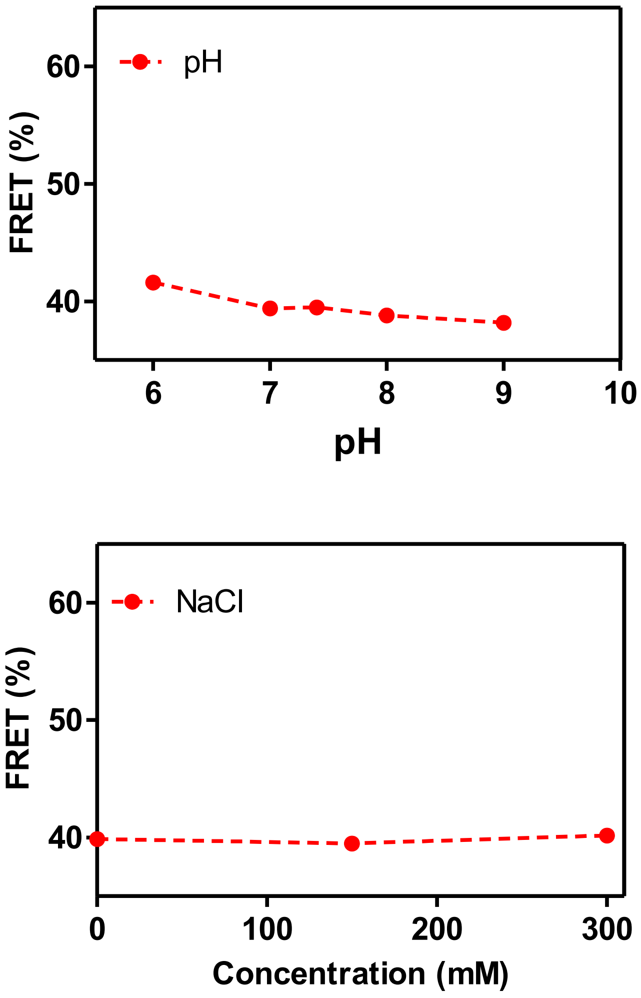

Figure S3. FRET efficiencies plotted against different pH or salt concentration. Error bars represent mean ± s.d.

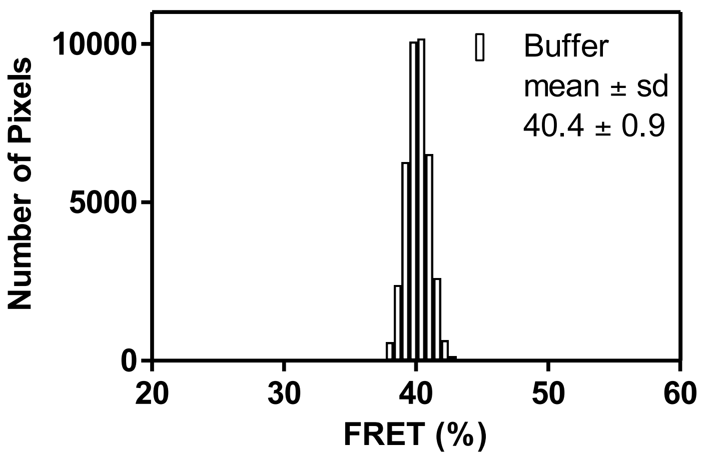

Figure S4. A pixel based evaluation was performed to calculate FRET for each pixel. The counts are plotted against FRET and the mean and standard deviation are calculated to illustrate the heterogeneity within a buffer measurement *in vitro* reflecting the experimental uncertainty.

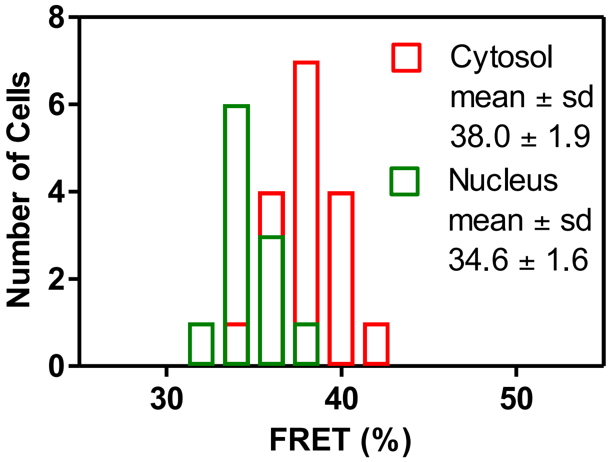

Figure S5. The mean of each cytosol or nucleus was calculated and is plotted against the number of counts. Mean and s.d. are calculated to illustrate the cell-to-cell variability.

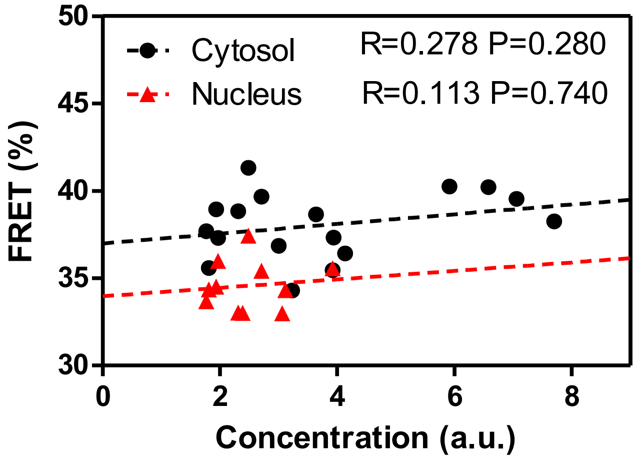

Figure S6. A relative concentration was calculated based on the used camera exposure and the intensity of direct acceptor excitation. The concentration was plotted against the average FRET. The correlation was calculated and R(Pearson) and respective P values are shown.

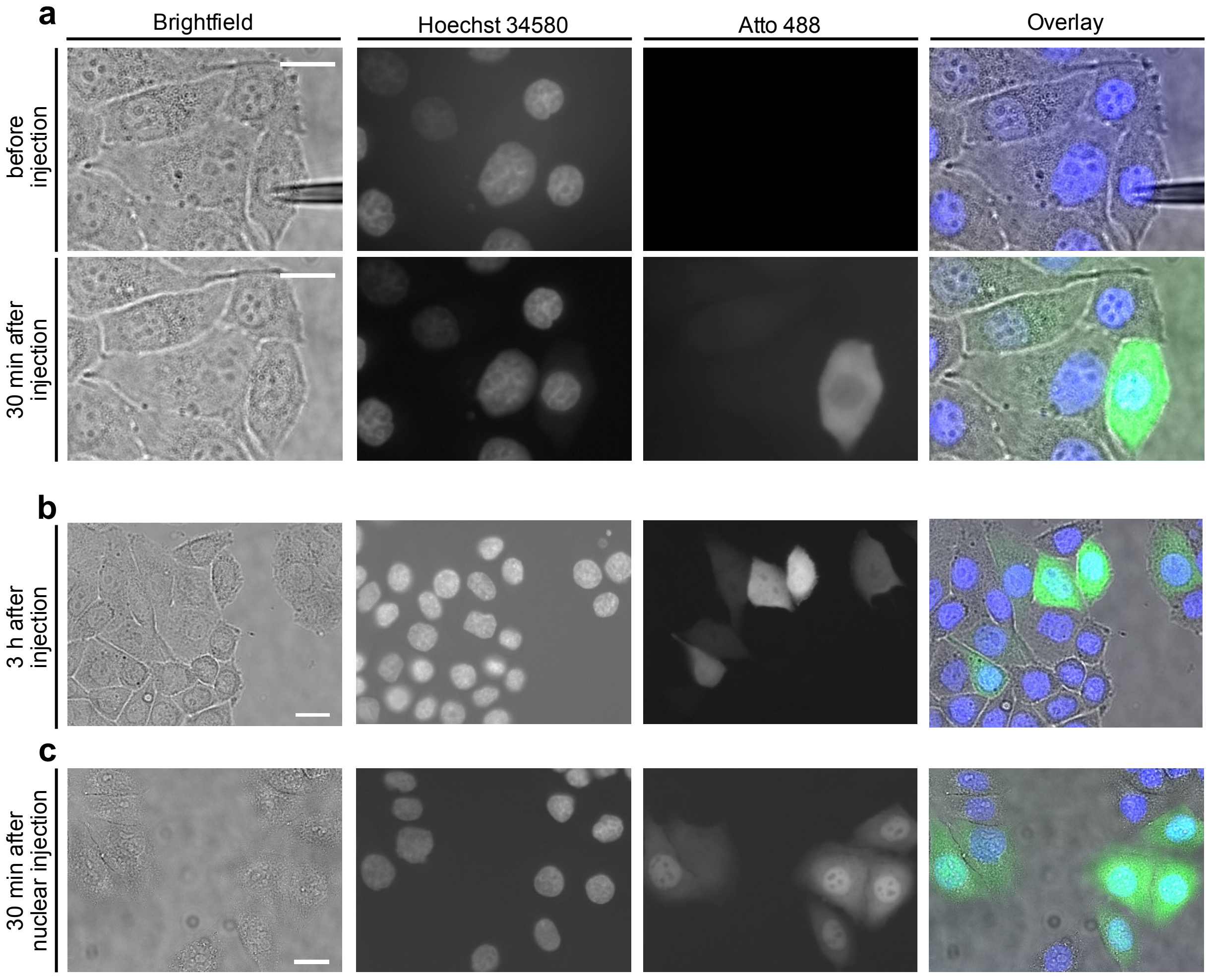

Figure S7. Microinjection controls showed unchanged cell morphology and nuclear integrity. **a**, Comparison of a HeLa cell before and 30 min after injection. Overlay: Hoechst 34580 (blue), brightfield image (gray), Atto 488 (green) **b**, Injected cells after 3 h incubation at RT. Overlay: Hoechst 34580 (blue), brightfield image (gray), Atto 488 (green). **c**, Nuclear injected cells after 30 min incubation at RT. Overlay: Hoechst 34580 (blue), brightfield image (gray), Atto 488 (green). Scale bars, 20 µm.

S.3 Supplement References

[1] a) G. Matthews, A. Colman, *Nucl. Acids Res.* **1991**, *19*, 6405-6412; b) J. Martin, F. Hartl, *Proc. Natl. Acad. Sci. USA* **1997**, *94*, 1107-1112.

[2] J. Feige, D. Sage, W. Wahli, B. Desvergne, L. Gelman, *Microsc. Res. Tech.* **2005**, *68*, 51-58.

[3] Z. Xia, Y. Liu, *Biophys. J.* **2001**, *81*, 2395-2402.

[4] a) S. N. Lim, N. A. Zeenathul, M. M. L. Azmi, *Pertanika J. Sci. & Technol.* **2011**, *19*, 273-283; b) M. J. Roberti, C. W. Bertoncini, R. Klement, E. A. Jares-Erijman, T. M. Jovin, *Nat. Methods* **2007**, *4*, 345-351; c) Y. Phillip, V. Kiss, G. Schreiber, *Proc. Natl. Acad. Sci. USA* **2012**, *109*, 1461-1466; d) Y. Zhang, L.-C. Yu, *Curr. Opin. Biotechnol.* **2008**, *19*, 506-510.

[5] K. Viigipuu, P. Kallio, *Altern Lab Anim.* **2004**, *32*, 417-423.
